# Strabismic Adults’ Experiences of Psychosocial Influence of Strabismus—A Qualitative Study

**DOI:** 10.1177/23779608241278456

**Published:** 2024-09-05

**Authors:** Anna Mason, Katja Joronen, Laura Lindberg, Marika Kajander, Nina Fagerholm, Anja Rantanen

**Affiliations:** 1 Faculty of Social Sciences, Health Sciences, 7840 Tampere University, Tampere, Finland; 2 HUS Helsinki University Hospital, Head and Neck Center, Helsinki, Finland; 3 Department of Nursing Science, 8058 University of Turku, Turku, Finland

**Keywords:** strabismus, psychosocial health-related quality of life, psychosocial well-being, nursing, qualitative research, inductive content analysis

## Abstract

**Introduction:**

Strabismus influences health-related quality of life. Individuals may have functional and psychosocial consequences of strabismus that impact their well-being. As strabismus is prevalent in all age groups, patients are seen in varied specialties in healthcare organizations. Therefore, healthcare professionals need to be aware of the psychosocial consequences when caring for strabismic individuals.

**Objective:**

To describe strabismic adults’ experiences of the psychosocial influence of strabismus.

**Methods:**

Twelve strabismic adults participated in face-to-face or online semistructured individual interviews to share their experiences of the psychosocial influence of strabismus. The participants were selected purposefully. The data were analyzed using inductive content analysis.

**Results:**

The participating adults described challenges with social environments, which showed stress in social situations and pressure in interactions. Strabismic adults had experienced staring from others, avoided social situations, and were uncomfortable with photography. They hid their strabismus, avoided eye contact, and struggled with intimate relationships. They also expressed struggles with mental well-being, which were emotional and psychological burdens. The adults described feeling negative emotions, worry, and irritation due to their condition. Struggles with self-confidence, difficulties with acceptance, negative thoughts about their appearance, experiences of bullying, and dealing with being different were described.

**Conclusion:**

Strabismic adults experience psychosocial consequences of strabismus, influencing their psychosocial health-related quality of life. Further studies should focus on how healthcare professionals could support strabismic individuals’ psychosocial well-being.

## Introduction

Strabismus is an eye condition whereby the eyes are misaligned; it is most often visible as one eye points in a different direction. The strabismic eye might turn in any direction or rarely rotate around its axis. The eye can be constantly or intermittently deviated, or the condition can alternate between the eyes. Worldwide, 3%–4% of adults have strabismus (Buffenn, 2021; Fieß et al., 2020; Hashemi et al., 2019; MacKenzie et al., 2016). Adults might have childhood-onset strabismus, or strabismus might be secondary to other conditions, such as thyroid eye disease, trauma, cranial nerve palsies, neurological diseases, or vision loss (Al-Omari et al., 2022). Surgical and nonsurgical treatments, such as glasses and prism lenses, are used to treat strabismus (Buffenn, 2021; MacKenzie et al., 2016).

Health-related quality of life (HRQOL) can be defined as one's well-being and functioning in the physical, mental, and social dimensions of life (Kaplan & Hays, 2022). Strabismus can influence all these dimensions. Blurred or double vision (diplopia), ocular discomforts, tiredness of the eyes, and abnormal head or body posture can affect physical function. The symptoms disrupt everyday life: watching TV, reading, cooking, and partaking in hobbies; driving may also be difficult (Kumaran et al., 2019; Wang et al., 2018). Strabismus can also influence interpersonal relationships, making one hide their strabismic eye and avoid social interactions. As the condition changes one's facial appearance, it can affect self-image, and bullying decreases mental and social well-being. People with strabismus may have symptoms of depression, negative emotions, and anxiety and struggle with self-perception and appearance-related issues (Ehlers et al., 2023; MacKenzie et al., 2016; Paduca et al., 2021; Wang et al., 2018). Due to the visibility of strabismus, employers might have misconceptions about persons living with strabismus, and the chance to obtain employment or promotion may be reduced, thus impacting one's economic well-being. Additionally, lower visual functioning is a risk for injuries; therefore, strabismus can be considered a public health concern (Buffenn, 2021; Wang et al., 2018).

## Review of the Literature

The Adult Strabismus Questionnaire (AS-20) can assess strabismic adults’ HRQOL. The AS-20 was developed by interviewing patients about their experiences living with strabismus (Hatt et al., 2009). The Finnish version of AS-20 is available for Finnish patients (Mason et al., 2023). Health-related quality of life is lower in strabismic compared to nonstrabismic adults, and strabismic women have lower overall and psychosocial HRQOL than strabismic men (Al-Omari et al., 2022).

Strabismus surgery generally increases one's HRQOL concerning physical and psychosocial well-being. Anxiety and depression levels have improved postoperatively (Ehlers et al., 2023). However, clinically successful surgery does not always improve psychosocial well-being; for some patients, preoperative psychosocial support could be helpful (MacKenzie et al., 2016). Individuals commonly seek surgery to improve their appearance, increase self-confidence, enhance social relationships, and receive better job opportunities (Al-Omari et al., 2022; Paduca et al., 2021). Often, patients with strabismus and surgery in childhood will require treatment in adulthood as the condition can reoccur (Al-Omari et al., 2022).

Nurses have an overarching role in the multidisciplinary team, providing care for patients in varied sectors of healthcare organizations (Lindahl Norberg & Strand, 2022). As people living with strabismus might present to nurse clinics in occupational, student health, or even a variety of specialties in hospitals, nurses should be aware and educated on how strabismus influences one's HRQOL, particularly in the psychosocial dimension, to improve care for all patients (Buffenn, 2021).

Previous international studies show that strabismus has psychosocial implications on HRQOL (Al-Omari et al., 2022; Ehlers et al., 2023; Wang et al., 2018). To further understand the consequences of strabismus, a qualitative study aiming to describe strabismic adults’ experiences of the psychosocial influence of strabismus was conducted.

## Methods

### Design

This study has a qualitative descriptive design, using semistructured interviews and inductive content analysis. A qualitative method was selected to explore and understand phenomena within this population (Holloway & Galvin, 2017; Kyngäs, 2019b). The COREQ checklist guided the reporting of the study.

### Participants and Setting

This study was conducted at a university hospital's ophthalmology outpatient clinic specializing in strabismus. The clinic cares for strabismic adults and children; in 2022, over 3000 outpatient visits and approximately 350 surgeries occurred.

Participants were recruited purposefully to reach the most informative participants who would share their experiences for the study (Moser & Korstjens, 2018). The study initially aimed to describe the experiences of the psychosocial influence of strabismus and then describe the patients’ expectations for psychosocial support. This article reports the experiences of the psychosocial influence of strabismus. Inclusion criteria were adult patients (≥18 years) attending the clinic with experiences of the psychosocial influence of strabismus. They had to be able to communicate in Finnish and have no severe psychiatric or neurological illnesses. Multiprofessional staff at the unit approached patients who had answered *sometimes, often, or always* on the Finnish AS-20 items 1–10 measuring psychosocial HRQOL (Mason et al., 2023) and offered to participate in this study. Participation was voluntary and did not affect care.

Altogether, 18 patients with visible strabismus were approached, of which 13 volunteered, received a written information leaflet, and signed a consent form. Five declined to participate due to their schedules or lack of interest. One did not reply to requests for an interview despite several attempts to contact them. Therefore, 12 adult patients were interviewed. Patients could choose whether to participate in an individual face-to-face or online interview. Eight interviews were held face-to-face at the clinic after the patient's appointment and four online on Microsoft Teams™ at a suitable time for the patients. With participants’ permission, the interviews were audio-recorded; three online interviews were video-recorded.

### Data Collection

The data were collected between August 2022 and February 2023 using a semistructured interview guide. The first author (AM) is a doctoral researcher in nursing science and conducted interviews independently as a full-time researcher. The interview guide was produced jointly with the research group. Neither the first author nor the research group knew the interviewees.

All the interviews commenced with the first author explaining the research objective, data security, confidentiality, and the previous and next steps of the PhD study. The first author ensured participation was voluntary and that the participants approved the recording. It was explained that the interviewer had a nursing background but was not a clinical expert on strabismus and did not participate in the clinical care of strabismic patients. However, due to their years of working in ophthalmology, the interviewer had developed an interest in studying the influence of strabismus on Finnish adults’ HRQOL. How the study's results could be used to further improve the services for strabismic patients was also explained.

Background questions included sex and age, which were categorized to maintain anonymity. The questions regarding strabismus concerned whether strabismus was in one or both eye(s), the onset of strabismus, and previous strabismus surgeries. Patients were asked to describe their experiences of how strabismus influenced their social well-being, such as interactions and social relationships and situations. Exploratory questions such as *you mentioned earlier… can you please tell me more… I understood you saying… did I understand that correctly… is there anything else you would like to tell me on this subject…* were used to deepen the knowledge of the experiences as guided by Holloway and Galvin (2017). Following the open-ended question on social well-being, the influence of strabismus on psychological well-being, such as mental well-being, feelings, and thoughts of self, was inquired. The same exploratory questions were used. All adults were interviewed once; the interviews lasted from 28 to 45 min. Field notes were written during the interviews. Data saturation—a point in data collection when no more new experiences were described—was achieved after ten interviews (Kyngäs, 2019b; Moser & Korstjens, 2018). However, two more interviews were conducted to ensure the data answered the research questions.

### Data Analysis

The data were analyzed using the inductive content analysis approach to gain a deeper understanding of the phenomenon, as the previous knowledge is deficient (Kyngäs, 2019a). The recordings were transcribed verbatim, and the transcriptions were checked by listening to the recordings. The transcriptions comprised 83 pages of text, including the following research questions: (1) How has strabismus affected your psychosocial well-being? and (2) What kind of psychosocial support do you expect from healthcare professionals? The second research question is reported in a separate article. The first author read the transcriptions several times to gain an understanding of the participants’ experiences. With the research objective clearly in focus, meaning units were chosen from the transcribed text to describe the strabismic adults’ experiences. Field notes and an interview diary strengthened the interpretations. Meaning units were condensed and then grouped to form subcategories of similar experiences. Several meaning units had more than one condensed meaning unit. Subcategories were given descriptive names and further connected to form categories. The research group discussed and reviewed the analysis at all stages (Kyngäs, 2019a). The meaning units were translated into English to report the results. Table 1 presents an example of the analysis.

**Table 1.** Example of the Analytical Process.

Meaning unit	Condensed meaning unit	Subcategory	Category	Main category
“I really hate to be photographed because strabismus can always be seen in the photos… that's why I avoid those situations.” ID 12	Hates to be photographed, as strabismus shows in the photos ID 12	Discomfort with being photographed	Stress in social situations	Challenges with the social environment
“If there have been any photography sessions, I went…but I have always felt that I don’t want to go anywhere where photos are taken.” ID 13	Does not want to go anywhere where photos are taken ID 13			
“With strangers… as in public places, people have more courage there, even to stare, compared to this room, as the situation is different, and people are not brave to stare here.” ID 20	Strangers are braver about staring in public places ID 20	Experiences of people staring		
“People stare… it is impossible not to notice.” ID 2	Staring from others is noticed ID 2			
“For sure, it is difficult for me to look people in the eye; I try to avoid it so the other would not notice my eye.” ID 19	Tries to avoid eye contact so others would not notice the eye ID 19	Avoiding eye contact	Pressure in interactions	
“I haven’t had the time to get used to it (strabismus), and I always avoid all eye contact.” ID 4	Always avoids all eye contact ID 4			
“And then… I am constantly fixing my eye. I blink and move my gaze—like I know when I look at you, my eye is playing its own game.” ID 20	Blinking so strabismus does not show ID 20	Hiding strabismus		
“When I look at myself on the computer screen and see my strabismus, I start to avoid… I might not look straight ahead or in the eye, but I try to look from an angle so that the strabismus would not show… It is not a pleasant situation.” ID 3	Tries to look from an angle to hide strabismus ID 3			

### Ethical Considerations

The Declaration of Helsinki was followed (WMA, 2013). Ethical and organizational approvals were acquired from the healthcare organization. Participants were given verbal and written information about the study before signing the consent form. Participation was voluntary. Patients were told they could stop their participation at any time; however, the information they share can be used in the study as per ethical and research permissions (Holloway & Galvin, 2017). Individuals were also informed they have a right to an appointment with a psychologist if they feel they need it.

For patient confidentiality, face-to-face individual interviews were held in hospital rooms with soundproof doors, and online meetings were arranged so no one else was in the interviewer's room. Individuals were told that their names had been replaced in the study with ID codes, and only the first author had access to the files connecting ID codes and names. All data were saved in the first author's hospital's secure database, following the organization's data policy. Recordings were deleted after being transcribed and checked. The research team discussed the findings to fully understand the phenomenon, and the first author returned to the transcriptions several times to confirm the participants’ experiences (Holloway & Galvin, 2017).

## Results

### Participant Characteristics

The participants (*n* = 12) were eight females and four males who sought care for strabismus. Most had childhood-onset strabismus (*n* = 9) and at least one previous surgery (*n* = 9). Table 2 reports the participants’ characteristics.

**Table 2.** Participants’ Characteristics.

Participant ID	Sex	Age categorized	Previous surgery	Onset of strabismus	Interview method	Duration (in minutes)
1	Female	18–30	1	Childhood	Online	35
2	Female	64–	0	Adult	Face-to-face	44
3	Male	45–63	3	Childhood	Online	40
4	Female	64–	1	Adult	Face-to-face	38
8	Female	45–63	1	Childhood	Face-to-face	34
9	Male	31–44	1	Childhood	Face-to-face	28
10	Male	45–63	0	Adult	Face-to-face	34
11	Male	18–30	2	Childhood	Face-to-face	36
12	Female	18–30	2	Childhood	Online	36
13	Female	18–30	3	Childhood	Face-to-face	35
19	Female	31–44	2	Childhood	Online	45
20	Female	18–30	1	Childhood	Face-to-face	44

**Figure 1.**
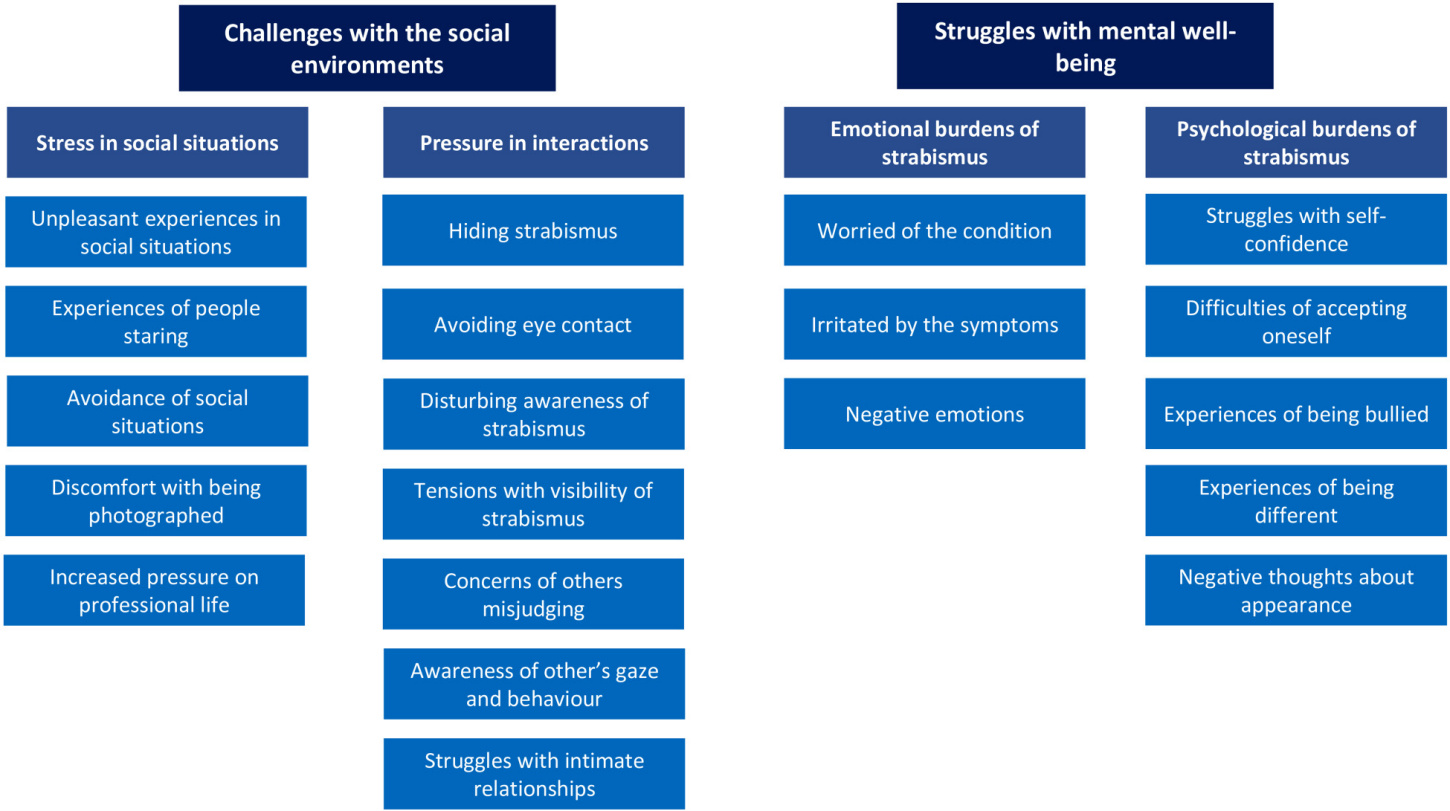
Experiences of psychosocial influence of strabismus.

The strabismic adults shared their experiences with strabismus; these experiences created challenges with their social environment and added to their mental health struggles. Results are presented in Figure 1 and described with the authentic quotations.

### Challenges with the Social Environments

#### Stress in Social Situations

Participants described having unpleasant experiences in social situations where people had looked past them or avoided looking at them. Situations when someone had asked about or commented on their eyes made them feel bad. Being in crowded places, such as on a bus, was stressful as people could easily notice their strabismus.

“Well, it is probably that… others do not look straight into my eye but avoid it altogether.” ID 9

They recounted experiences of people staring at them. They felt others were very interested in their eyes and generally tried to stare discreetly but failed. They also suggested that people stare because they do not know about the condition.

“Some people stare for long and don’t seem to be able to stop staring; it makes me so uncomfortable.” ID 20

Individuals avoided social situations due to their eyes, stating that staying home rather than facing the reactions of strangers was easier for avoiding misunderstandings. With strabismus, the eye can look like it is pointing in a different direction than where the strabismic person is looking, which can create misinterpretation or even conflict in social situations.

“I don’t go to all places because I am worried there will be a misunderstanding, as my eye points somewhere else, which causes me stress.” ID 11

Being photographed was uncomfortable. Participants recounted childhood and adolescent experiences from school photography sessions and how uncomfortable they had felt as strabismus showed easily in the photos. Recently, they felt challenged by friends continuously updating their social media with selfies.

“All the school photos—I did not like them at all. I had to think about hiding the strabismus by keeping my head bit sideways… but it was there… always, everyone will remember me like this. It was stressful; I don’t think many with strabismus like having photos taken.” ID 13

Strabismus negatively influenced professional life. Participants described their experiences of work- or study-related presentations when they had the expertise and knowledge to speak, but pondering the visibility of strabismus made the situation awkward and stressful. Individuals working in customer service expressed that strabismus had sometimes caused conflicts with customers, as the customers felt the agent was not looking at them but past them.

“In giving presentations at work, I feel awkward as I have to constantly think about whether my strabismus is showing.” ID 3

#### Pressure in Interactions

Patients described avoiding eye contact in interactions, as they were aware of strabismus and did not want to cause confusion. Symptoms of strabismus, such as diplopia, also made maintaining eye contact difficult. They hoped that after corrective surgery, they could look people straight in the eye. They explained that if the person they interact with knows the situation, they try to maintain eye contact.

“Now, when I know what this situation—this interview—is all about, I can look this way straight at you… But when I don't know, I avoid eye contact, so the condition does not show.” ID 2

The patients explained hiding their strabismus so others would not see it. Practical examples included scratching, rubbing, blinking, or closing the strabismic eye, looking down or somewhere else, standing a particular way, tilting their head, looking sideways, and always turning their whole body rather than just their head. Some planned their seating at social gatherings in a way so they would not be opposite strangers. They expressed that although hiding is mostly an automatic act, it takes energy in interactions.

“When I speak in a big group, well, I can’t turn my gaze towards the person because I get a feeling… because then the person I am speaking to sees my strabismus… So I have to turn my whole body towards the person so they cannot see it.” ID 1

Strabismus could be felt continuously; thus, it was constantly in their thoughts throughout the day. It could not be forgotten or ignored as it bothered the participants, thus impacting interactions.

“This is so visible… It is present all the time… I can't forget it and ignore it… Everybody sees it, and I can feel it constantly.” ID 4

Interviewees thought of the visibility of their strabismus often and expressed that it might create tension in interactions, making them seem more reserved and unlike themselves when connecting with people. During interactions, particularly when meeting new people, they expressed wondering greatly if the other person noticed their strabismus, what they thought of their eyes, how they reacted to their eyes, or whether they felt tension during the interaction. They also felt that discussing strabismus with new people was difficult; if they did mention it, their new acquaintances would notice their condition.

“Well, it is always on my mind, and I think of what the other person thinks of me when my eyes are like this.” ID 13

Interviewees described feelings of others misjudging them and thinking of them as weird, stupid, or unintelligent because of their eyes. They felt that people judged them and looked down on them as if strabismus made them unequal.

“Sometimes, I feel that during an interaction, the other person thinks I am weird because of my eye and looks down on me—like I am not equal to them. Yes, this is my feeling, but their body language reveals this, and their facial expressions do, too.” ID 11

Participants explained that they read other people's behavior and focused on their eyes to assess where the other is looking, as they did not want their strabismus to make the other person uncomfortable. However, they saw how people looked at their eyes and quickly realized how the other person's behavior changed when the condition was noticed.

“I am aware… in the interaction, I follow the other's eyes closely… particularly because they are looking at me… These are the things I am aware of, but I feel that others do not really understand.” ID 12

Strabismus was considered a struggle in intimate relationships. Finding a life partner was more difficult, as potential partners might fear the condition. First dates caused additional pressure, as the participants wondered if their strabismus should be mentioned before the date or if the date noticed their condition from the photo. Some felt their partner was sometimes ashamed of their appearance and feared their eyes had been why their previous relationships had not flourished.

“Well, yes, it is… finding a partner has been challenging.. Well, hmm, maybe they fear the other one looks so different—in appearance, I mean.” ID 9

### Struggles with Mental Well-Being

#### Emotional Burdens of Strabismus

The patients feared their strabismus worsening or not knowing why their eye had deteriorated. Availability and accessibility of the treatment options caused concerns, and they recalled feeling occasional anxiety about their eyes.

“So, the kind of worry I have… can strabismus get even worse than it is now… or can I receive help… and is there even help for my eyes… or do I have to manage with just one eye?” ID 10

Strabismus symptoms irritated the participants. They described difficulties with eye fatigue, the inability to work long periods on the computer, and the need for several different glasses for different situations.

“Sometimes, it is so annoying…with those glasses… I have ten different pairs of glasses and must think about what I shall wear now… oh, I can’t watch TV now—now I have to cover my eye… it is a hassle.” ID 4

Having strabismus provoked negative emotions such as feeling upset, embarrassed, shame, and psychological pain. Feeling upset was occasional, particularly when they were alone or reliving memories from adolescence when strabismus had caused them to feel bad about themselves. Depressive thoughts and anxiety were infrequent and undiagnosed. Embarrassment and shame were connected to social situations with others and the inability to work in the profession they trained for.

“Being off work is so embarrassing and shameful… I can’t work due to the symptoms of strabismus, and I feel embarrassment and shame because of it.” ID 8

#### Psychological Burdens of Strabismus

Participants struggled with self-confidence. They mentioned feeling inadequate, particularly in puberty, to others with nonstrabismic eyes. Self-confidence was not developed during adolescence due to misalignment, impacting their choices for further education. Lack of self-confidence in adolescence caused self-criticism and sometimes manifested as self-disruptive behavior. Self-confidence was also influenced by the lack of the general public's understanding of strabismus, as adults living with strabismus felt they had to prove themselves more than others without the condition.

“Yes, I feel that strabismus has impacted my studying… probably mostly due to self-confidence, which I feel has not developed as it should have. So, I have many issues… I was interested in studying a particular field. I would have loved to go to the university and get a degree, but I feel I am not enough for this or that.” ID 19

Acceptance of oneself with misaligned eyes was reported as an ongoing process that is not yet complete. Participants mentioned living with poor eyesight was preferable than coping with strabismus and that life would be better if their eyes were straightened. As strabismus cannot always be corrected by surgery or other treatments, acceptance felt very difficult.

“Well, I have to all the time… continuously work in my mind that I am good and am okay with the way I am. It does not matter… I feel that sometimes I have managed this well, but it is such hard work… so hard.” ID 13

The interviewees had experienced bullying, mostly in childhood and in adulthood. They were called ugly names—weird, stupid, and stupid-looking—mostly at school. In adulthood, they had received comments from dating apps or others in interactions. Bullying had crushed their self-confidence, causing upset, and further psychological implications.

“But, yes. Once I received feedback. Someone I know said this to me.. Yuck! Why are your eyes like that?… No one will ever want you… and then I really collapsed and cried every day.” ID 1

They also described feeling different from others; for example, in childhood, they were the only ones with misaligned eyes in their year. Now, as adults, they were the only ones with strabismus in their social and professional circles. As they could not look straight, they were different. They described their first time at the clinic as surprisingly positive, as they had seen others with the same condition.

“I had not met anyone who also had strabismus… so I had thought that because I am the only one with strabismus in my hometown, I am different. And then I came here and saw others with strabismus and realized I am not the only one… it felt good.” ID 2

Strabismic adults thought negatively about their appearance and expressed that their eyes made them look less attractive. Younger or female participants mentioned this in particular. Others had told them not to worry about their eyes and appearance but as appearance mattered to them, not worrying was impossible.

“I always talk about my eye—that it is crossed. I suffer so much from it… For me appearance has always been important… therefore, I am so bothered by this crooked eye.” ID 4

## Discussion

This study aimed to describe strabismic adults’ experiences of the psychosocial influence of strabismus. As the phenomenon is not widely studied, qualitative research with semistructured interviews was conducted.

Participants recalled stress in social situations, such as unpleasant experiences and people staring, causing them to avoid social gatherings and interactions. They also described how uncomfortable they found being photographed and wanted to avoid those situations, as strabismus is visible in photos. These results strengthen Wang et al.'s (2018) previous findings. The adults expressed avoiding eye contact and hiding their strabismus to prevent others from seeing their eyes, which could be due to previous unpleasant experiences in social situations, as MacKenzie et al. (2016) indicated. Asymmetry of the eyes could also be interpreted as unfriendliness or impoliteness (Wang et al., 2018), which could explain why the participants of the current study avoided eye contact and social situations and hid their strabismic eye. The interviewees expressed concerns about others misjudging them and thinking of them as being less intelligent. Estes et al. (2020) discuss public self-consciousness and one's belief in how others perceive them. Their research shows that strabismus surgery improves patients’ public self-consciousness and reduces social anxiety. This indicates that people living with strabismus suffer from what others might think of them, which may generate social anxiety (Estes et al., 2020). The current study participants described struggles in intimate relationships due to their eyes, aligning with Wang et al.'s (2018) previous study and the reasons for strabismic adults’ seeking surgical treatment as Paduca et al. (2021) and Al-Omari et al. (2022) reported.

Strabismus emotionally burdened the patients who described fearing their condition worsening or treatment being unavailable. Education of healthcare personnel and increased general awareness of strabismus can relieve these concerns. Previous research states that adults can delay seeking help due to many misconceptions about strabismus, and correct education can reduce these misconceptions and the delay in accessing services (Al-Omari et al., 2022; Paduca et al., 2021; Wang et al., 2018).

Negative emotions were common, as Wang et al. (2018) reported previously. The strabismic adults described feeling upset about the situation and occasionally felt depressive feelings and anxiety. However, these occasional feelings were not assessed on a measure, such as the Hospital Anxiety and Depression Scale, which Ehlers et al. (2023) used for their study. In the current study, as by Wang et al. (2018), the interviewees also expressed embarrassment, shame of their eyes and the situation.

Strabismus affects adults’ self-confidence in many ways. This aligns with Paduca et al.'s (2021) study, where improved self-confidence was one motivator for young adults and adults to seek surgical treatment. Many recounted experiences of bullying in the current study. Although most of the experiences were in childhood, a few had been exposed to mean comments as adults. Bullying had caused psychological implications, concurring with previous studies (Buffenn, 2021; Wang et al., 2018). Most childhood-onset strabismus develops before age 10, and the difference in appearance can cause negative attitudes and social bias from others (Huang & Pineles, 2023). As adults recounted bullying at schools, school staff should be aware of bullying due to strabismus, and education on strabismus should be provided for the staff and pupils.

Participants expressed negative thoughts on their appearance, as they felt they were unattractive. Wang et al.'s (2018) study also described these experiences, although the cultural context differs. Strabismus alters appearance by changing the symmetry of the eyes (Buffenn, 2021; Wang et al., 2018). Therefore, providing surgical treatment for strabismic adults can reconcile patients’ appearance to match society's expectations of normal (Estes et al., 2020), easing the feelings of being different and diminishing the negative thoughts about their appearance. However, for some strabismic adults, surgery does not improve psychosocial well-being (MacKenzie et al., 2016); thus, preoperative psychosocial support is needed (Ehlers et al., 2023).

## Strengths and Limitations

Criteria of trustworthiness in a qualitative study are defined as credibility, transferability, dependability, and confirmability (Korstjens & Moser, 2018). This study was conducted by interviewing patients who had experienced psychosocial hindrances of strabismus and were willing and able to share their subjective experiences. Inclusion criteria and purposeful recruitment enabled identifying and approaching persons with experience on the psychosocial influence of strabismus. A doctoral researcher with previous interviewing experience conducted the interviews. The participants are described, and their authentic quotations are used in the results, enhancing the study's credibility and transferability. Dependability is supported by describing details of data collection and analysis. Additionally, confirmability is improved by constant reflection, self-awareness, and discussions throughout the process with the research team (Korstjens & Moser, 2018). These strengthen the study.

The results are experiences of 12 strabismic individuals, and they are not generalizable to all people living with strabismus. This is a common limitation of qualitative studies. However, increasing awareness of the psychosocial influence of strabismus to improve patient-centered care is important. It is worth noting that dependability would have been increased by involving participants in evaluating findings, which was impossible in this study (Korstjens & Moser, 2018). Transferability to other strabismic patients would have been improved even further by reporting participants’ AS-20 scores and the clinical data, such as diplopia and the direction and amount of strabismus. Future studies need to observe this.

## Implications for Practice

This study's results can be used to educate healthcare professionals on the psychosocial consequences of strabismus and provide support for strabismic patients of all ages. Support for psychosocial well-being should be available for people living with strabismus.

## Conclusions

Strabismic adults experience psychosocial influence of strabismus, both with challenges in their social environment and struggles with mental well-being. Stress in social situations, pressure in interactions, and the emotional and psychological burdens of strabismus influence adults’ HRQOL. As participating adults reflected many experiences from their childhood and adolescence, it is imperative to understand how strabismus influences psychosocial well-being in children and adolescents and provide timely support for their psychosocial health. Health and social care professionals working in childhood, adolescent, and adult services, including educational environments, should understand the psychosocial challenges of people living with strabismus to support and guide them toward available care. Additionally, these results can increase the general societal awareness of strabismus.

## Supplemental Material

sj-pdf-1-son-10.1177_23779608241278456 - Supplemental material for Strabismic Adults’ Experiences of Psychosocial Influence of Strabismus—A Qualitative Study Supplemental material, sj-pdf-1-son-10.1177_23779608241278456 for Strabismic Adults’ Experiences of Psychosocial Influence of Strabismus—A Qualitative Study by Anna Mason, Katja Joronen, Laura Lindberg, Marika Kajander, Nina Fagerholm and Anja Rantanen in SAGE Open Nursing
